# Response to Interferon-Beta Treatment in Afro-Caribbeans with Multiple Sclerosis

**DOI:** 10.1155/2011/950126

**Published:** 2011-05-23

**Authors:** S. Jeannin, R. Deschamps, N. Chausson, P. Cabre

**Affiliations:** Department of Neurology, Pierre Zobda-Quitman Hospital, Fort-de-France, 97261 Martinique, French West Indies, France

## Abstract

*Background*. Multiple sclerosis (MS) patients of African ancestry have a more aggressive disease course than white patients and could be resistant to interferon-beta (INFB). *Methods*. We studied the impact of INFB in treatment-naive Afro-Caribbean (AC) with clinically definite MS using our European Database for Multiple Sclerosis (EDMUS) (2003–2010). Main outcome measures were annual relapse rate after 2 years of treatment, proportion of exacerbation-free subjects 48 weeks after initiating INFB, and time to first relapse. *Results*. 76 AC-MS (59F/17M) were identified. Annual relapse rate of 1.29 decreased to 0.83 (−35.6%) after 2 years of treatment. The proportion of relapse-free patients at 48 weeks was 46.2%. Median time to first relapse was 52 weeks. *Conclusion*. INFB is not strong enough to control AC-MS patients in many cases which is problematic in a population of worse MS prognosis.

## 1. Introduction

Subjects of African ancestry have a more aggressive course of multiple sclerosis (MS) than white patients [1–5]. In addition, they could be less responsive to interferon-beta (INFB) than Caucasians [6]. We aimed to determine the effect of INFB in a large sample of Afro-Caribbean (AC) MS patients.

## 2. Methods

All MS patient visits at the Fort de France Department of Neurology have been entered prospectively in the European Database for Multiple Sclerosis (EDMUS) since 2003. Data included sex, age at onset, date of INFB initiation and discontinuation, type of INFB, date and characteristics of relapses, and Expanded Disability Status Scale (EDSS) score. Patients are seen when INFB is initiated, at months 1, 2, 3, and 6 and then twice a year, as requested by French Drug Agency. Data from patients with clinically definite MS [7] having received INFB as first-line therapy and for any length of time were extracted from the Fort de France EDMUS database (August 31, 2010). Those with age between 18 and 55 years, EDSS of 0 to 5.5, and at least 2 relapses in the 2 previous years before INFB-treatment initiation were eligible for the study. Data taken in consideration were age at onset, disease duration at the beginning of treatment, annualized relapse rate for the 2 years prior to INFB therapy and for the first two years on INFB therapy, and EDSS when treatment was initiated as well as at 2 years of treatment. Patients who remained free from relapses at 24 weeks, 48 weeks, and 104 weeks of INFB therapy were identified. A Relapse was defined as the appearance of new neurological symptoms or worsening of pre-existing symptoms lasting at least 24 hours, preceded by 30 days of clinical stability, accompanied by objective change on examination (worsening of 0.5 point on the EDSS or 1.0 point on the motor, cerebellar, brainstem, or visual functional system scores) in the absence of fever (pseudorelapse). Relapses were treated with intravenous methylprednisolone. Subjects who did not confirm diagnosis of clinically definite MS and patient who fulfilled revised diagnostic criteria for neuromyelitis optica were excluded [8].

### 2.1. Statistical Analysis

Kaplan Meier curve was used to assess median time to first relapse and Log-rank test to evaluate the effect of categorical variables. The level of statistical significance was set at *P* < .05. Statistical analysis was conducted using StatView statistical version 5.0 software.

## 3. Results

We identified 76 AC-MS patients fulfilling inclusion criteria (Table 1): 59 females and 17 males (sex ratio F/M: 3.5). Of these, 50% received Betaseron (Shering AG, Berlin, Germany) 8 MIU every other day, 21.1% Rebif (Serono, Geneva, Switzerland) 44-*μ*g three times weekly, and 28,9% Avonex (Biogen, Cambridge, Mass, USA) 30-*μ*g each week. The mean age at onset of MS was 30.1 years, and the mean disease duration at initiation of INFB therapy was 4.7 years. Mean duration of treatment was 38.3 months (median: 34). In the two years preceding INFB therapy, mean number of relapses was 2.3 giving a mean annualized relapse rate of 1.29 that decreased to 0.83 (−35.6%) after 2 years of treatment. Mean EDSS at baseline was 2.52 (median 2.5) and slightly increased to 2.66 at two years followup (*P* = .16).

Patients who remained free from relapses at 24 weeks, 48 weeks, and 104 weeks of INFB therapy were 66,6%, 46.2%, and 29.2%, respectively (7 relapse-free patients who received treatment for < 6 months were excluded because a period of < 6 months of therapy would be too short to evaluate a clinical treatment effect). The median time to first relapse was 12 months (95% Confidence Interval: 10.7–13.3 months). One variable trended to significance was identified to contribute to early failure of INFB therapy in AC-MS: men had a shorter time to first relapse than women (11 months versus 14 months; *P* = .08). Age at onset of MS, disease duration, EDSS at baseline, relapse rate before treatment, and type of INFB did not contribute. 

## 4. Discussion

In our observational cohort of AC-MS subjects, mean disease duration, mean age at first symptom, and annualized mean number of relapses 2 years before INFB reproduce inclusion criteria of pivotal and head-to-head trials of INFB. INFB therapy reduced the relapse rate by 35.6% in this series. This is within the expected range of treatment (48 to 53%) effects reported in pivotal trials of INFB [9–11], but it may still not good enough for most patients. The annualized rate of relapses over 2 years remained high (0.83) for ours patients treated with either INFB-1a or INFB-1b. By comparison, the annualized relapse rate at end of two years study was 0.7 and 0.5 in INFB-1a and INFB-1b arms, respectively, in INCOMIN trial [12], 0.54 and 0.65 in INFB-1a 44 *μ*g and INFB 30 *μ*g arms, respectively, in EVIDENCE trial [13], and 0.61 in INFB-1a pivotal trial [11]. The proportion of patients who remained relapse-free at 48 weeks after initiating INFB therapy was 46.2%. Likewise, percentage of relapse-free patients at two years (29.2%) in our cohort is outside the range of INFB (32 to 51%) effects in PRISMS [10], INCOMIN trials [12]. Time to first relapse was 12 months in our cohort, shorter to that observed in INFB-1a 44 *μ*g arm (13.5 months) of the EVIDENCE trial. However, time to first relapse was 47.3 weeks in INFB-1a pivotal trial [11]. They are some of limitations to our observational study. First, we are not able to discuss primary endpoint of pivotal trials of INFB because there is no control group in our study. Second, disease activity on MRI scans was not available. Finally, we cannot exclude an overestimation of relapses during INFB therapy by nonblinded neurologists. However, our data showed a poorer response to INFB in AC-MS patients concerning all secondary clinical endpoints in pivotal and head-to-head trials of INFB. Furthermore, our results reproduce those provided in Afro-American (AA) MS by EVIDENCE post-hoc analysis. In this trial, a similar proportion (47% versus 46.2%) of relapse-free AA-MS patients after 48 weeks of treatment was observed (Table 1). We only found a trend for male gender to predict early INFB therapy failure. Others showed that in the setting of daily MS practice, Caucasian INFB responders had an older age, a higher relapse rate prior to treatment, and longer disease duration at the time INFB was initiated [14]. Poor beneficial outcome of INFB therapy in people of African ancestry has not been investigated but could result either alone or in combination with development of neutralizing antibodies against INFB or polymorphisms in genes interplaying with INFB response such as glypicans (GPC5), hyaluronan proteoglycan link protein (HAPLN1), interferon receptors (INFAR1 and INFAR2), and MxA [15]. Given highly aggressive nature of disease in MS patients of CA descent and suboptimal response to INFB, consideration should be given to upgrading therapy to more aggressive options (monoclonals antibodies or immunosuppressors) early on in the disease course.

## Figures and Tables

**Table 1:** Demographic/clinical analysis of AC-MS, WA-MS, and AA-MS of EVIDENCE study.

Clinical/demographic Information	AC-MS *n* = 76	WA-MS (EVIDENCE) *n* = 616	AA-MS (EVIDENCE) *n* = 36
Female, %	77.6	74.5	86.1

Mean age at first symptom, years (range)	30.1 (14–50)	31.8 (18–55)	32.1 (18–55)

Mean disease duration, years (median)	4.7 (2)	6.9	5.6

Mean treatment duration, weeks	153.2	62	62

Mean EDSS at treatment (median)	2.52 (2.5)	2.3	2.8

Mean number of relapses in the 2 previous years (median)	2.3 (2)	2.6	2.5

%, relapse free at 48 weeks	46.2	57	47
